# Pathogenesis of Preeclampsia: The Genetic Component

**DOI:** 10.1155/2012/632732

**Published:** 2011-12-01

**Authors:** Francisco J. Valenzuela, Alejandra Pérez-Sepúlveda, María J. Torres, Paula Correa, Gabriela M. Repetto, Sebastián E. Illanes

**Affiliations:** ^1^Departamento de Obstetricia and Ginecología y Laboratorio de Biología de la Reproducción, Universidad de Los Andes, Santiago 7620001, Chile; ^2^Centro de Genética Humana, Facultad de Medicina, Clínica Alemana-Universidad del Desarrollo, Santiago 7620001, Chile

## Abstract

Preeclampsia (PE) is one of the main causes of maternal and fetal morbidity and mortality in the world, causing nearly 40% of births delivered before 35 weeks of gestation. PE begins with inadequate trophoblast invasion early in pregnancy, which produces an increase in oxidative stress contributing to the development of systemic endothelial dysfunction in the later phases of the disease, leading to the characteristic clinical manifestation of PE. Numerous methods have been used to predict the onset of PE with different degrees of efficiency. These methods have used fetal/placental and maternal markers in different stages of pregnancy. From an epidemiological point of view, many studies have shown that PE is a disease with a strong familiar predisposition, which also varies according to geographical, socioeconomic, and racial features, and this information can be used in the prediction process. Large amounts of research have shown a genetic association with a multifactorial polygenic inheritance in the development of this disease. Many biological candidate genes and polymorphisms have been examined in their relation with PE. We will discuss the most important of them, grouped by the different pathogenic mechanisms involved in PE.

## 1. Introduction

Preeclampsia (PE) and its complications and associated pathologies have become one of the main causes of maternal and fetal morbidity and mortality in the world, causing nearly 40% of births delivered before 35 weeks of gestation. Moreover, PE has been strongly associated with an increased risk of later-life death due to cardiovascular disease, independent of other risk factors [1–3]. PE is present in around 5–10% of all pregnant women worldwide and despite the amount of resources invested in the research and treatment of this pathology, its development is still barely predictable and thus challenging to prevent and manage clinically. 

PE constitutes a clinical spectrum that includes “maternal” PE and “placental” PE [4]. In general, placental PE involves an abnormal placentation in a healthy woman, and in maternal PE there is a normal placentation in a woman with a preexistent pathology, like cardiovascular disease, chronic arterial hypertension, or diabetes. Nevertheless, in practice, the great majority of patients who will develop PE have both types to different degrees, and rather than being two distinct types of disease, they are the extremes of the same pathologic entity. These 2 origins may explain the variability in the severity and gestational age of presentation of this syndrome. Placental PE, with a poor trophoblastic perfusion, generates oxidative stress, with liberation of trophoblast factors to maternal circulation, which will induce a secondary inflammatory response and endothelial dysfunction [5]. In maternal PE, an inflammatory response takes place, involving all the inflammatory components of circulation, including the endothelium [5]. 

The understanding of the underlying factors that explain the pathogenesis of PE and the early identification of the patients at risk of the disease will help in the development of preventative or early therapeutic interventions, aimed to reduce the associated morbidity and mortality during pregnancy, but also the long-term severe problems that PE may produce or is associated with.

## 2. Pathogenesis of PE: A Placental Originated Disease

Despite the breakthroughs in the understanding of the pathogenesis of PE, the mechanisms that finally trigger the disease are still not clearly elucidated. Nevertheless, it seems clear that the development of PE during pregnancy requires the presence of the placenta, given that this clinical syndrome will not be developed if it is not present, and it disappears soon after placental delivery [6]. In placental PE, it is also widely accepted that the physiopathological process of PE begins with inadequate trophoblast invasion early in pregnancy, which produces an increase in oxidative stress contributing to the development of systemic endothelial dysfunction in the later phases of the disease, leading to the characteristic clinical manifestation of PE, with hypertension, proteinuria, and edema.

During normal placentation, the cytotrophoblast cells form a highly invasive extravillous trophoblast (EVT) that can migrate into the decidua and invade the first third of the myometrium, inducing the remodelling of spiral arterioles to produce the low-resistance vascular system that allows a 10-fold increase in blood flow, essential for fetal growth [7–9]. The relative reduction of uteroplacental flow, secondary to abnormal placentation caused by an impaired trophoblast invasion [8], is the trigger for the development of PE [10–12]. This was first highlighted by the histopathological findings in the site of placental implantation, where 80 to 100% of patients with PE have a deficit of the physiological invasion of the maternal spiral arteries by the EVT [8, 13]. It has been postulated that the physiological changes that favor the invasive phenotype of these cells are due to the exposure of cytotrophoblast cells to a hypoxic environment. The normal concentration of oxygen in the first trimester placenta is only about 3% O_2_ (±18 mm Hg) and this low oxygen milieu is believed to facilitate trophoblast invasion [14]. In the normal placentation process, the cytotrophoblast invasion is regulated by the gradient of oxygen concentration between the placenta and maternal arteries. Therefore, the hypoxic environments that face the cytotrophoblast at the beginning of the placentation change gradually to a normoxic environment as invasion takes place [15]. In pathologies with an abnormal placentation, the trophoblastic invasion is poor and limited only to spiral arteries present in superficial decidua. The mechanisms involving this poor placentation are still areas of research.

In summary, the events that lead to the development of a PE may be explained by a first stage of defective trophoblastic invasion, which occurs early in pregnancy, with uteroplacental circulation remaining in a state of high resistance during pregnancy, which can be detected by an increased resistance of the uterine arteries [16]. The persistence of a state of underperfusion produces placental hypoxia and local oxidative stress, resulting in a systemic inflammatory response and endothelial dysfunction, leading to the onset of the clinical symptoms of PE [4]. The first stage is difficult to diagnose, while the second is the clinical syndrome itself. 

## 3. Prediction of PE

Numerous methods have been used to predict the onset of PE with different degrees of efficiency. These methods have used fetal/placental and maternal markers in different stages of pregnancy in order to predict the disease. The fetal/placental markers could be divided in (1) trophoblast invasion (PLGF, IGFBP-1, PAPP-A, Doppler ultrasound, and HLA-G), (2) placental hypoxia (sFlt-1, VEGF, PLGF), (3) reactive oxygen species (lipid peroxide), and (4) placental function (activin/inhibin, CRH/CRHBP, and PAI-2). The maternal markers that have been used could be classified as (1) metabolic syndrome (BMI, Leptin, insulin, and glucose), (2) endothelial function (PAI-1, Fibronectin, VCAM/ICAM), (3) prooxidants (8-epi-PGF2a), (4) antioxidant reserve (vitamins C and E), and (5) immune function (AT-R autoantibodies) [17]. 

Despite the improvements in the prediction of the disease, there is no treatment that reverses this pathology once it has begun. This is mainly explained by the fact that the different tests used to predict PE have a better performance after the first trimester of pregnancy, a period in which the intervention to decrease the prevalence of the disease has proved to be ineffective [18]. This is why more research is needed to develop clinical tools that allow a prediction in even more early stages, or even before the patient gets pregnant. Moreover, it should be noted that the PE has a clear genetic component and each of the etiological factors that are involved in its pathogenesis, immune maladaptation, placental ischemia, or oxidative stress may have a genetic implication [19].

## 4. The Genetic Component

From an epidemiological point of view, many studies have shown that PE is a disease with a strong familial predisposition, which also varies according to geographical, socioeconomic, and racial features. It has been reported that women with first-degree relatives with PE have 5 times more risk of developing the disease, while those with second-degree relatives have their risk doubled [20, 21]. Moreover, it is believed that paternal genes also play an important role in the development of PE. This is evidenced by the increased risk of PE in women with pregnancies of men who have previously been involved in pregnancies complicated with PE [22, 23]. This is of special importance since genomic imprinting results in involvement of paternal genes in the control of invasion and placental growth, whereas maternal genes inhibit it and are responsible for the adaptive immune response of pregnancy [24]. A large genetic association study of PE was published by Goddard et al. [25] that reported a study evaluating 775SNPs in 190 genes in more than 350 PE mother and offspring pairs and 600 control pairs. They detected six genes with a significative maternal-fetal genotype interaction related to PE in *IGF1*, *IL4R*, *IGF2R*, *GNB3*, *CSF1*, and *THBS4*. These findings and others suggest a multifactorial polygenic inheritance with a genetic component in the development of this disease [26, 27]. 

Many biological candidate genes and polymorphisms have been examined in its relation with PE. We will discuss some of them, grouped by the different pathogenic mechanisms involved in PE (see Table 1).

### 4.1. Immune Maladaptation

#### 4.1.1. ERAP1 and 2

The endoplasmic reticulum aminopeptidases 1 and 2 (ERAP1 and 2) are important in the immune response in terms of the antigen presentation [51] and they are colocalized within the endoplasmic reticulum (ER). The process starts with a proteolysis by the proteosome in the cytosol, and finally N-extended peptides are processed by aminopeptidase to mature the epitope which is presented by MHC class I. Data in mice have shown that ERAP1 trims MHC class I presented peptides in vivo and is the major trimming enzyme in the ER lumen; however ERAP2 also plays a role in the trimming of proteins [52]. In a murine model, it has been demonstrated that ERAP1 can be secreted in response to LPS/INF*γ* and activate macrophages in culture [53]. In human placenta, aminopeptidase RNA of ERAP1 has been detected by RT-PCR [54]. Recent studies in Australian/New Zealand and Norwegian populations have shown that *ERAP2* SNPs (p.392 K>N and p.669 L>Q; rs2549782 and rs17408150, resp.) are associated with PE susceptibility [28]. Recently, these SNPs have been also associated with increased risk of hypertensive disorders in pregnancy in African American population, but not in a Chilean one [55], supporting the idea that PE has a heterogeneous basis with variation between different ethnic group.

#### 4.1.2. TNFSF13B

TNFSF13B is a member of the tumor necrosis factor family of ligands. *TNFSF13B* is localized in chromosome region 13q32-q34 and is implicated in the regulation of immune response to infections, autoimmune disease, and inflammation. In the third trimester of pregnancy, human placenta expresses TNFSF13B in villous cytotrophoblast cells (CT) and mesenchymal cells from villous core (MC). The receptors for TNFSF13B are expressed in both tissues [56, 57]. It has been proposed that the role of TNFSF13B [56] may be to modulate the immune system of the mother or to help in the development of fetal immune system. At the placental level, it is believed that TNFSF13B has an antiapoptotic effect [56]. Recently, Fenstand et al. [29] have shown a genetic variation of *TNFSF13B*, which is correlated with the susceptibility of PE. In his study, these authors detected three rare SNPs in *TNFSF13B* (rs16972194, rs16972197, and rs56124946) in Australian/New Zealand families with PE, but not in Norwegian ones. These results suggest that TNFSF13B could contribute to normal immunological adaptation during pregnancy, and the presence of these SNPs contributes to an abnormal placentation in some populations. 

#### 4.1.3. HLA-G Gene Polymorphism

This gene, localized in 6p21.3, is member of HLA class I molecule and has immunosuppressive properties. This characteristic has been implicated in the modulation of the maternal immune system, giving a possible inhibiting role during pregnancy when the mother gets in contact with the fetus [30]. HLA-G polymorphism is associated with recurrent spontaneous abortion and PE. Moreau et al. [30] analyzed several polymorphisms in placenta of pregnant women with PE and controls, finding a higher frequency of G*0106 HLA-G polymorphism in preeclamptic placentas compared with control (21.2% and 6.6%, resp.), suggesting a modulator effect of *HLA-G* over pregnancy and the risk of PE.

### 4.2. Vascular and Endothelial Function

#### 4.2.1. VEGF

The VEGF is a family with seven members, VEGF-A, VEGF-B, VEGF-C, VEGF-D, VEGF-E, VEGF-F, and PIGF. VEGF-C is expressed mainly in the heart, placenta, ovary and embryonic tissues [58]. The receptors of VEGF named VEGFR-1/Flt-1, VEGFR-2/Flk-1 and VEGFR-3/Flt-4 are VEGF tyrosine kinase receptors. VEGFR-1 has a much weaker kinase activity and modulates the endothelial cell cycle. VEGFR-2 is the more important receptor for VEGF in VEGF-induced mitogenesis and permeability. VEGFR-3 is expressed in lymphatic endothelial cells and participates in mitosis, migration, differentiation, and survival cells [58]. Several polymorphisms in VEGF have been correlated with an increased risk of PE. Polymorphisms in VEGF-A have been implicated in the risk of PE, and down regulation of VEGF and VEGFR expression has been also reported in patients with severe PE [59, 60].

A recent study using an array for 50,000 genecentric SNPs identified 124 SNPs in 6 genes related to angiogenesis. This data indentified allelic variation of *VEGF-C*, *Flt-1*, and *Flt-4* from 606 women (489 African American and 117 white Caucasian). Two *VEGF-C* SNPs (rs1485766 and rs6838834) were associated with PE in African American women and one *VEGF-C* SNP (rs7664413) was associated with PE in Caucasian women [31]. This author also highlights the association between *Flt-1* polymorphism and PE, showing that two *Flt-1* SNPs (rs12584067 and rs7335588) are associated with PE in black women and other two, *Flt-1* SNP (rs722503) and *Flt-4* (rs307826), are more prevalent in white patients with PE [31]. However, some data have shown that some polymorphism of VEGF can be protective for PE. In a Hungarian cohort of nulliparous patients, a study looking for the *VEGF* r. 405 C > polymorphism has found that patients with the *VEGF* 405 G allele have less severity of PE [61]. Nevertheless, a recent study [62] showed that the *VEGF* 405 G allele (rs2010963) is not associated with PE in a Mexican population of pregnant women. These differences highlight the variability and implications of the presence of some SNPs in diverse ethnic groups. 

Finally, Shim et al. [32] have characterized another polymorphism of *VEGF* in Korean pregnant women using PCR and restriction fragment length polymorphism assay. The *VEGF* +936 C>T has been located in the 3′-unstranslated region (UTR), and it has been associated with PE. However, the small number of patients in this study precludes making more general conclusions.

#### 4.2.2. eNOS

The NO is synthesized by endothelial nitric oxide synthase (eNOS, NOS3) using L-arginine as a substrate. NO is the endothelium-derived relaxing factor and has a crucial role in the regulation of smooth muscle tone in the vascular system [63], being a critical element for the correct blood perfusion of the placenta. Recently, it has been reported that the activity of eNOS is reduced in patients with PE [33]. Several polymorphisms have been described for eNOS, but two of them have been strongly related with PE, SNP G c.894 G>T, which encodes an amino acid substitution in eNOS (p.Glu298Asp) and −786 T>C polymorphism. A study in 844 Colombian pregnant women reported that patients homozygous for Asp298 (894 T) allele have more risk of PE than women with Glu298 allele [64]. Similarly, an important correlation has been reported between hypertension and Asp298 genotype in pregnant women of Japan [65].

#### 4.2.3. CYP11B2

The steroid 11/18-beta-hydroxylase is encoded by *CYP11B2* gene that is located in 8q24.3. The protein is physically localized in mitochondrias of the zone glomerulosa of the adrenal cortex, synthesizing the mineralocorticoid aldosterone [66]. The −344 C/T polymorphism within 5′regulatory region of *CYP11B2* disrupts a putative steroidogenic factor-1 site, and the homozygosity for SF-1 T/T variant has been reported as a protective factor against the risk of PE. In contrast, the heterozygous state is not protective of PE [34]. Moreover, −344 C/T polymorphism has a strong linkage disequilibrium respect intron 2 polymorphisms in *CYP11B2* and contributes to hypertension in subjects with a raised aldosterone-to-renin ratio [35].

### 4.3. Thrombophilic Disorders

Thrombophilic conditions are associated with an increased risk of venous thromboembolic events during pregnancy; however, there is still controversy as to whether they may adversely affect other pregnancy outcomes such as PE [67]. Women with PE have levels outside the normal range for fibronectin and von Willebrand factor, that are markers of endothelial cell injury [68], and recently several polymorphisms of genes coding for vascular proteins have been associated with PE. 

#### 4.3.1. The human Prothrombin (F2)

The gene is localized in chromosome 11p11-q12, and the increase of prothrombin during pregnancy can neutralize the effect of physiologic hemodilution, increasing blood viscosity and the likelihood of thromboembolic events [68]. Recently Seremak-Mrozikiewicsz et al. [36] have shown in Polish population that a polymorphism of prothrombin (G20210A) could be associated with an increased risk of developing severe PE.

#### 4.3.2. Factor V Gene

The gene is localized in the 1q23 implicated in several diseases. Several studies, have suggested an important correlation between factor V Leiden SNPs (1691 G>A) and the risk of severe PE [37, 38] and a recent meta-analysis has confirmed these findings [69]. The study of Seremak-Mrozikiewicsz [36] also showed that 1691 G>A polymorphism contained at least one variant allele A (GA and AA) in the group of women with severe and mild PE.

#### 4.3.3. SERPINE1 Gene

PE is associated with thrombosis of the intervillous space of the placenta. The *SERPINE1* gene encodes endothelial plasminogen activator inhibitor-1 (PAI-1), the major inhibitor of fibrinolysis (member of the serine protease inhibitor family). Yamada et al. [39] assessed the association between PE and the 4 G/5 G polymorphism of the *PAI1* gene in 115 PE patients, 210 pregnant controls, and 298 healthy volunteer controls. The frequency of homozygotes for the 4 G allele was significantly higher in the patients than in the control pregnant women or healthy volunteers. The 4 G allele frequency was also significantly higher in patients than in the control groups. However, other studies have not found the same association [19].

### 4.4. Metabolism and Oxidative Stress

#### 4.4.1. PON-1

The paraoxonase-1 (*PON-1*) is a member of a three-gene family (*PON-2* and *PON-3*), and is located in chromosome 7 (q21.22). The protein, primarily synthesized in the liver, has a release into the circulation and is associated with high-density lipoproteins (HDLs). The principal action of PON-1 is the protection of acute toxicity and oxidative stress involved in the development of atherosclerosis. PON-1 also has a role in the hydrolysis of organophosphate insecticides and nerve agents [70, 71]. There is evidence that shows that preeclamptic women have lower levels of serum HDL, higher level of serum triglycerides, and lower level of serum Apo-A1 than control women [72], and this has been correlated with lower PON-1 serum activity [73]. A study in 3266 Caucasian women who were randomly selected from 23 British towns investigated the relation between *PON-1* p.Q192R polymorphism and hypertension. They did not find evidence of an increased risk of hypertension during pregnancy in carrier patients, but an association was seen with preterm birth. This polymorphism could reduce the hydrolysis and modify the redox equilibrium in the maternal blood [74]. A relation between *Pon-1* p.Q192R and serum oxidized LDL levels has been also reported [72]. Finally, Isbilen et al. [75] reported a differential distribution of *PON-1* p.Q192R and L55M polymorphism between PE and normal patients. The authors detected high levels of homocysteine concentration in preeclamptic women when homozygotes for 192RR and 55RR were present.

Iron-induced oxidation and free radical attack of the R-SH group of different maternal plasma molecules like to aminothiols, including cysteine, homocysteine, and cysteinylglycine result in the formation of radical disulfides. These changes produce disequilibrium in the redox thiol status, which is displaced to a higher oxidized state [17]. The level of maternal plasma homocysteine is higher in severe PE than in mild PE and control groups. Elevated levels of maternal homocysteine increase the risk for endothelial dysfunction and atherosclerosis and occlusive vascular disorders [76].

### 4.5. MTHFR, MTRR, and MTR Genetic Polymorphisms

Decrease in MTHFR protein levels or activity by different gene variants induces increased levels of homocysteine [40–42]. The most extensively studied variant in *MTHFR* gene is the polymorphism *MTHFR* g.677C>T (rs1801133), which changes an alanine to valine in aminoacid 222 (p.A222V) in the enzyme regulatory domain and causes a thermolabile enzyme with decreased activity at 37°C and so hyperhomocysteinemia [40–43]. This reduced activity of *MTHFR* due to *MTHFR* g.677C>T polymorphism may impair the remethylation pathway [44]. The frequencies for the T-allele/TT-genotype of *MTHFR* g.677C>T polymorphism are variable in different populations, being 0,11/0,00 in African Americans and 0,59/0,35 in Mexicans, respectively [45]. *MTHFR* A1298C is another common polymorphism and it produces the change of glutamate to alanine in aminoacid 429 (Glu429Ala) within the enzyme catalytic domain [40, 46, 47]. In general, the frequency of CC genotype is about 0.1 and C allele frequency is about 0.36 [42]. As with *MTHFR* g.677C>T, *MTHFR* A1298C polymorphism has been reported to be associated with altered methionine-homocysteine metabolism (MHM) and increased levels of homocysteine [41]. Polymorphisms in *MTHFR* gene and hyperhomocysteinemia have been associated with recurrent pregnancy loss, gestational hypertension, placental abruption and PE [40, 43].

The most common polymorphism in the *MTRR* gene is g.66A>G (rs1801394) substitution, changing isoleucine to methionine in aminoacid 22 (I22M). *MTRR* g.66A>G polymorphism has been associated with reduced activity of MTRR enzyme resulting in hyperhomocysteinemia and altering the methylation of DNA [42, 43, 47]. It has also been observed that the coexistence of the *MTHFR* g.677C>T genotype with the *MTRR* g.66A>G polymorphism may exacerbate the effect of the *MTHFR* variant alone [46].

A common polymorphism in *MTR* is g.2756A>G substitution, which results in an amino acid change of an aspartic acid to a glycine (p.D919 G), at the penultimate position in a long helix that leads out of the cobalamin domain. Having the glycine residue at this position could have an effect on the secondary structure of the protein and therefore have functional consequence [48–50]. Recently, Furness et al. [50] have evaluated one-carbon metabolism enzyme polymorphisms in patients with uteroplacental dysfunction, suggesting that the maternal and fetal *MTR* g.2756A>G allele represents an important risk factor for the development of uteroplacental insufficiency, which includes PE and intrauterine growth restriction [50]. The potential negative effect of combined polymorphisms of the *MTHFR*, *MTR*, and *MTRR* genes on plasma homocysteine levels in at-risk population needs further investigation.

## 5. Conclusion

PE is a common disease in pregnancy worldwide, causing substantial short- and long-term morbidity for the newborn and the mother. PE is a multifactorial disease, where maternal and fetal factors converge to result in a multicomponent risk. No single factor has been identified as capable of determining the disease, and several are needed to trigger symptoms of PE.

Various studies in different populations have identified maternal polymorphisms associated with PE through candidate gene approaches. These findings will need to be complemented by currently available genome-wide approaches, evaluation of interaction between genes, genes and environment, and the contribution of paternal and embryonic genotypes. Further prospective studies will be necessary to assess the predictive potential of markers identified through these and other strategies.

Despite substantial advances in understanding the pathogenesis of PE, the development of simple tests to identify individuals or populations at risk still remains a substantial research, epidemiologic, and clinical challenge.

## Figures and Tables

**Table 1:** Overview of the different polymorphism described.

Gene	Function	SNP	Reference
*ERAP 1-2*	Aminopeptidases 1-2. Related to immune antigen presentation	p.392 K>N and p.669L>Q	[28]

*TNFSF13B*	Regulation of immune response, member of tumor necrosis factor family	rs16972194 rs16972197 rs56124946	[29]

*HLA-G*	Member of HLA class I molecule and has immunosuppressive properties	G*0106 *HLA-G *	[30]

*VEGF*	Modulates the cell cycle, migration, and differentiation	rs1485766 rs6838834 rs7664413 rs2010963 +936 C>T	[31, 32]

*Flt-1*	FMS-related tyrosine kinase. It shows tyrosine protein kinase activity	rs12584067 rs7335588 rs722503	[31]

*eNOS*	Endothelial nitric oxide synthase	p.Glu298As-786T>C	[33]

*CYP11B2*	Steroid 11/18-beta-hydroxylase	−344C/T	[34, 35]

*Prothrombin (F2)*	Increases blood viscosity and the likelihood of thromboembolic events	G20210A	[36]

*Factor V *	Coagulation factor V	1691G>A	[36–38]

*SERPINE1 *	Endothelial plasminogen activator inhibitor-1 (PAI-1), the major inhibitor of fibrinolysis	4 G/5 G polymorphism	[39]

*MTHFR*	Methylenetetrahydrofolate reductase	rs1801133 A1298C	[40–46]

*MTRR*	Methionine synthase reductase	rs1801394	[40–43, 46, 47]

*MTR*	Methyltetrahydrofolate-homocysteine S-methyltransferase	g.2756A>G	[48–50]

SNP: single nucleotide polymorphism.

## References

[1] Bellamy L, Casas JP, Hingorani AD, Williams DJ (2007). Pre-eclampsia and risk of cardiovascular disease and cancer in later life: systematic review and meta-analysis. *British Medical Journal*.

[2] McDonald SD, Malinowski A, Zhou Q, Yusuf S, Devereaux PJ (2008). Cardiovascular sequelae of preeclampsia/eclampsia: a systematic review and meta-analyses. *American Heart Journal*.

[3] Mongraw-Chaffin ML, Cirillo PM, Cohn BA (2010). Preeclampsia and cardiovascular disease death: prospective evidence from the child health and development studies cohort. *Hypertension*.

[4] Ness RB, Roberts JM (1996). Heterogeneous causes constituting the single syndrome of preeclampsia: a hypothesis and its implications. *American Journal of Obstetrics & Gynecology*.

[5] Borzychowski AM, Sargent IL, Redman CWG (2006). Inflammation and pre-eclampsia. *Seminars in Fetal and Neonatal Medicine*.

[6] Redman CW (1991). Current topic: PE and the placenta. *Placenta*.

[7] Aplin JD (1991). Implantation, trophoblast differentiation and haemochorial placentation: mechanistic evidence *in vivo* and *in vitro*. *Journal of Cell Science*.

[8] Brosens IA, Robertson WB, Dixon HG (1972). The role of the spiral arteries in the pathogenesis of preeclampsia. *Obstetrics & Gynecology Annual*.

[9] Whitley GSJ, Cartwright JE (2010). Cellular and molecular regulation of spiral artery remodelling: lessons from the cardiovascular field. *Placenta*.

[10] Page EW (1939). The relation between hydatid moles, relative ischaemia of the gravid uterus and the placental origin of eclampsia. *American Journal of Obstetrics & Gynecology*.

[11] Rauramo I, Forss M (1988). Effect of exercise on placental blood flow in pregnancies complicated by hypertension, diabetes or intrahepatic cholestasis. *Acta Obstetricia et Gynecologica Scandinavica*.

[12] Burton GJ, Yung HW, Cindrova-Davies T, Charnock-Jones DS (2009). Placental endoplasmic reticulum stress and oxidative stress in the pathophysiology of unexplained intrauterine growth restriction and early onset PE. *Placenta*.

[13] Khong TY, De Wolf F, Robertson WB, Brosens I (1986). Inadequate maternal vascular response to placentation in pregnancies complicated by pre-eclampsia and by small-for-gestational age infants. *British Journal of Obstetrics and Gynaecology*.

[14] Rodesch F, Simon P, Donner C, Jauniaux E (1992). Oxygen measurements in endometrial and trophoblastic tissues during early pregnancy. *Obstetrics & Gynecology*.

[15] Genbacev O, Zhou Y, Ludlow JW, Fisher SJ (1997). Regulation of human placental development by oxygen tension. *Science*.

[16] Albaiges G, Missfelder-Lobos H, Lees C, Parra M, Nicolaides KH (2000). One-stage screening for pregnancy complications by color Doppler assessment of the uterine arteries at 23 weeks’ gestation. *Obstetrics & Gynecology*.

[17] Lyall F, Belfort B (2007). *Pre-eclampsia Etiology and Clinical Practice*.

[18] Fayyad AM, Harrington KF (2005). Prediction and prevention of preeclampsia and IUGR. *Early Human Development*.

[19] Mütze S, Rudnik-Schöneborn S, Zerres K, Rath W (2008). Genes and the preeclampsia syndrome. *Journal of Perinatal Medicine*.

[20] Marshall Graves JA (1998). Genomic imprinting, development and disease—is pre-eclampsia caused by a maternally imprinted gene?. *Reproduction, Fertility and Development*.

[21] Ros HS, Lichtenstein P, Lipworth L, Cnattingius S (2000). Genetic effects on the liability of developing pre-eclampsia and gestational hypertension. *American Journal of Medical Genetics*.

[22] Haig D (1996). Gestational drive and the green-bearded placenta. *Proceedings of the National Academy of Sciences of the United States of America*.

[23] Roberts CT (2010). IFPA award in placentology lecture: complicated interactions between genes and the environment in placentation, pregnancy outcome and long term health. *Placenta*.

[24] Dekker G, Robillard PY, Roberts C (2011). The etiology of preeclampsia: the role of the father. *Journal of Reproductive Immunology*.

[25] Goddard KAB, Tromp G, Romero R (2007). Candidate-gene association study of mothers with pre-eclampsia, and their infants, analyzing 775 SNPs in 190 genes. *Human Heredity*.

[26] Trogstad L, Skrondal A, Stoltenberg C, Magnus P, Nesheim BI, Eskild A (2004). Recurrence risk of preeclampsia in twin and singleton pregnancies. *American Journal of Medical Genetics A*.

[27] Carreiras M, Montagnani S, Layrisse Z (2002). Preeclampsia: a multifactorial disease resulting from the interaction of the feto-maternal HLA genotype and HCMV infection. *American Journal of Reproductive Immunology*.

[28] Johnson MP, Roten LT, Dyer TD (2009). The *ERAP2* gene is associated with preeclampsia in Australian and Norwegian populations. *Human Genetics*.

[29] Fenstad MH, Johnson MP, Roten LT (2010). Genetic and molecular functional characterization of variants within *TNFSF13B*, a positional candidate preeclampsia susceptibility gene on 13q. *PLoS ONE*.

[30] Moreau P, Contu L, Alba F (2008). *HLA-G* gene polymorphism in human placentas: possible association of *G**0106 allele with preeclampsia and miscarriage. *Biology of Reproduction*.

[31] Srinivas SK, Morrison AC, Andrela CM, Elovitz MA (2010). Allelic variations in angiogenic pathway genes are associated with preeclampsia. *American Journal of Obstetrics & Gynecology*.

[32] Shim JY, Jun JK, Jung BK (2007). Vascular endothelial growth factor gene +936 C/T polymorphism is associated with preeclampsia in Korean women. *American Journal of Obstetrics & Gynecology*.

[33] Williams PJ, Pipkin FB (2011). The genetics of pre-eclampsia and other hypertensive disorders of pregnancy. *Best Practice and Research: Clinical Obstetrics and Gynaecology*.

[34] Escher G, Cristiano M, Causevic M (2009). High aldosterone-to-renin variants of *CYP11B2* and pregnancy outcome. *Nephrology Dialysis Transplantation*.

[35] Lim PO, Macdonald TM, Holloway C (2002). Variation at the aldosterone synthase (*CYP11B2*) locus contributes to hypertension in subjects with a raised aldosterone-to-renin ratio. *Journal of Clinical Endocrinology and Metabolism*.

[36] Seremak-Mrozikiewicz A, Drews K, Wender-Ozegowska E, Mrozikiewicz PM (2010). The significance of genetic polymorphisms of factor v leiden and prothrombin in the preeclamptic polish women. *Journal of Thrombosis and Thrombolysis*.

[37] Lin J, August P (2005). Genetic thrombophilias and preeclampsia: a meta-analysis. *Obstetrics & Gynecology*.

[38] Faisel F, Romppanen EL, Hiltunen M (2004). Susceptibility to pre-eclampsia in Finnish women is associated with R485K polymorphism in the factor V gene, not with Leiden mutation. *European Journal of Human Genetics*.

[39] Yamada N, Arinami T, Yamakawa-Kobayashi K (2000). The 4G/5G polymorphism of the plasminogen activator inhibitor-1 gene is associated with severe preeclampsia. *Journal of Human Genetics*.

[40] Mtiraoui N, Zammiti W, Ghazouani L (2006). Methylenetetrahydrofolate reductase C677T and A1298C polymorphism and changes in homocysteine concentrations in women with idiopathic recurrent pregnancy losses. *Reproduction*.

[41] Sharma P, Senthilkumar RD, Brahmachari V (2006). Mining literature for a comprehensive pathway analysis: a case study for retrieval of homocysteine related genes for genetic and epigenetic studies. *Lipids in Health and Disease*.

[42] Gos M, Szpecht-Potocka A (2002). Genetic basis of neural tube defects: II. Genes correlated with folate and methionine metabolism. *Journal of Applied Genetics*.

[43] Kluijtmans LAJ, Young IS, Boreham CA (2003). Genetic and nutritional factors contributing to hyperhomocysteinemia in young adults. *Blood*.

[44] Dasarathy J, Gruca LL, Bennett C (2010). Methionine metabolism in human pregnancy. *American Journal of Clinical Nutrition*.

[45] Bermúdez M, Briceño I, Gil F, Bernal J (2006). Homocysteine and polymorphisms of cystathionine synthase and methylentetrahydrofolate reductase in a healthy population from Colombia. *Colombia Medica*.

[46] Vaughn JD, Bailey LB, Shelnutt KP (2004). Methionine synthase reductase 66A→G polymorphism is associated with increased plasma homocysteine concentration when combined with the homozygous methylenetetrahydrofolate reductase 677C→T variant. *Journal of Nutrition*.

[47] Hague WM (2003). Homocysteine and pregnancy. *Best Practice and Research: Clinical Obstetrics and Gynaecology*.

[48] Drennan CL, Huang S, Drummond JT, Matthews RG, Ludwig ML (1994). How a protein binds B12: a 3.0 Å X-ray structure of B12-binding domains of methionine synthase. *Science*.

[49] van der Put NMJ, Eskes TKAB, Blom HJ (1997). Is the common 677C→T mutation in the methylenetetrahydrofolate reductase gene a risk factor for neural tube defects? A meta-analysis. *QJM*.

[50] Furness DLF, Fenech MF, Khong YT, Romero R, Dekker GA (2008). One-carbon metabolism enzyme polymorphisms and uteroplacental insufficiency. *American Journal of Obstetrics & Gynecology*.

[51] Georgiadou D, Hearn A, Evnouchidou I (2010). Placental leucine aminopeptidase efficiently generates mature antigenic peptides in vitro but in patterns distinct from endoplasmic reticulum aminopeptidase 1. *Journal of Immunology*.

[52] York IA, Brehm MA, Zendzian S, Towne CF, Rock KL (2006). Endoplasmic reticulum aminopeptidase 1 (ERAP1) trims MHC class I-presented peptides in vivo and plays an important role in immunodominance. *Proceedings of the National Academy of Sciences of the United States of America*.

[53] Goto Y, Ogawa K, Hattori A, Tsujimoto M (2011). Secretion of endoplasmic reticulum aminopeptidase 1 is involved in the activation of macrophages induced by lipopolysaccharide and interferon-*γ*. *Journal of Biological Chemistry*.

[54] Nagase T, Ishikawa KI, Miyajima N (1998). Prediction of the coding sequences of unidentified human genes. IX. The complete sequences of 100 new cDNA clones from brain which can code for large proteins *in vitro*. *DNA Research*.

[55] Hill LD, Hilliard DD, York TP (2011). Fetal *ERAP2* variation is associated with preeclampsia in African Americans in a case-control study. *BMC Medical Genetics*.

[56] Langat DL, Wheaton DA, Platt JS, Sifers T, Hunt JS (2008). Signaling pathways for B cell-activating factor (BAFF) and a proliferation-inducing ligand (APRIL) in human placenta. *The American Journal of Pathology*.

[57] Phillips TA, Ni J, Hunt JS (2003). Cell-specific expression of B lymphocyte (APRIL, BLyS)- and Th2 (CD30L/CD153)-promoting tumor necrosis factor superfamily ligands in human placentas. *Journal of Leukocyte Biology*.

[58] Hoeben A, Landuyt B, Highley MS, Wildiers H, Van Oosterom AT, De Bruijn EA (2004). Vascular endothelial growth factor and angiogenesis. *Pharmacological Reviews*.

[59] Lyall F, Greer IA (1996). The vascular endothelium in normal pregnancy and pre-eclampsia. *Reviews of Reproduction*.

[60] Zhou Y, McMaster M, Woo K (2002). Vascular endothelial growth factor ligands and receptors that regulate human cytotrophoblast survival are dysregulated in severe preeclampsia and hemolysis, elevated liver enzymes, and low platelets syndrome. *The American Journal of Pathology*.

[61] Bányász I, Bokodi G, Vásárhelyi B (2006). Genetic polymorphisms for vascular endothelial growth factor in perinatal complications. *European Cytokine Network*.

[62] Garza-Veloz I, Castruita-De la Rosa C, Cortes-Flores R (2011). No association between polymorphisms/haplotypes of the vascular endothelial growth factor gene and preeclampsia. *BMC Pregnancy and Childbirth*.

[63] Thuillez C, Richard V (2005). Targeting endothelial dysfunction in hypertensive subjects. *Journal of Human Hypertension*.

[64] Serrano NC, Casas JP, Díaz LA (2004). Endothelial NO synthase genotype and risk of preeclampsia: a multicenter case-control study. *Hypertension*.

[65] Kobashi G, Yamada H, Ohta K, Kato E, Ebina Y, Fujimoto S (2001). Endothelial nitric oxide synthase gene (*NOS3*) variant and hypertension in pregnancy. *American Journal of Medical Genetics*.

[66] Sewer MB, Waterman MR (2003). ACTH modulation of transcription factors responsible for steroid hydroxylase gene expression in the adrenal cortex. *Microscopy Research and Technique*.

[67] Battinelli EM, Bauer KA (2011). Thrombophilias in pregnancy. *Hematology/Oncology Clinics of North America*.

[68] Myatt L, Webster RP (2009). Is vascular biology in preeclampsia better?. *Journal of Thrombosis and Haemostasis*.

[69] Dudding T, Heron J, Thakkinstian A (2008). Factor V Leiden is associated with pre-eclampsia but not with fetal growth restriction: a genetic association study and meta-analysis. *Journal of Thrombosis and Haemostasis*.

[70] Costa LG, Giordano G, Furlong CE (2011). Pharmacological and dietary modulators of paraoxonase 1 (*PON1*) activity and expression: the hunt goes on. *Biochemical Pharmacology*.

[71] Draganov DI, Teiber JF, Speelman A, Osawa Y, Sunahara R, La Du BN (2005). Human paraoxonases (*PON1*, *PON2*, and *PON3*) are lactonases with overlapping and distinct substrate specificities. *Journal of Lipid Research*.

[72] Kim YJ, Park H, Lee HY (2007). Paraoxonase gene polymorphism, serum lipid, and oxidized low-density lipoprotein in preeclampsia. *European Journal of Obstetrics Gynecology and Reproductive Biology*.

[73] Uzun H, Benian A, Madazli R, Topçuoğlu MA, Aydin S, Albayrak M (2005). Circulating oxidized low-density lipoprotein and paraoxonase activity in preeclampsia. *Gynecologic and Obstetric Investigation*.

[74] Lawlor DA, Gaunt TR, Hinks LJ (2006). The association of the *PON1* Q192R polymorphism with complications and outcomes of pregnancy: findings from the British Women’s Heart and Health cohort study. *Paediatric and Perinatal Epidemiology*.

[75] Isbilen E, Yilmaz H, Arzu Ergen H, Unlucerci Y, Isbir T, Gurdol F (2009). Association of paraoxonase 55 and 192 gene polymorphisms on serum homocysteine concentrations in preeclampsia. *Folia Biologica*.

[76] Acilmis YG, Dikensoy E, Kutlar AI (2011). Homocysteine, folic acid and vitamin B12 levels in maternal and umbilical cord plasma and homocysteine levels in placenta in pregnant women with pre-eclampsia. *Journal of Obstetrics and Gynaecology Research*.

